# Randomized, Double-Blind, Placebo-Controlled Study of Anti-Mycobacterial Therapy (RHB-104) in Active Crohn’s Disease

**DOI:** 10.3390/antibiotics13080694

**Published:** 2024-07-25

**Authors:** David Y. Graham, Saleh A. Naser, Thomas Borody, Zbigniew Hebzda, Harry Sarles, Scott Levenson, Robert Hardi, Tomasz Arłukowicz, Petar Svorcan, Reza Fathi, Aida Bibliowicz, Patricia Anderson, Patrick McLean, Clara Fehrmann, M. Scott Harris, Shuhong Zhao, Ira N. Kalfus

**Affiliations:** 1 Departments of Medicine, Molecular Virology, and Microbiology, Michael E. DeBakey VA Medical Center and Baylor College of Medicine, Houston, TX 77030, USA; 2 Burnett School of Biomedical Sciences, University of Central Florida, Orlando, FL 32816, USA; saleh.naser@ucf.edu; 3 Center for Digestive Diseases, Sydney 2046, Australia; thomas.borody@cdd.com.au; 4 Specjalistyczne Centrum Medyczne Unimedica, 31-271 Krakow, Poland; zbigniew.hebzda@o2.pl; 5 Digestive Health Associates of Texas (DHAT) Research Institute, Garland, TX 75044, USA; harry.sarles@dhat.com; 6 Digestive Care Associates, Inc., San Carlos, CA 94070, USA; scottlevenson@sbcglobal.net; 7 Department of Gastroenterology, George Washington University Medical School, Washington, DC 20052, USA; drroberthardi@gmail.com; 8 Collegium Medicum, University of Warmia and Mazury, 10-082 Olsztyn, Poland; tomasz.arlukowicz@uwm.edu.pl; 9 School of Medicine, University of Belgrade, 11000 Belgrade, Serbia; svorcanp@mts.rs; 10 Zvezdara University Medical Center, 11000 Belgrade, Serbia; 11 RedHill Biopharma, Ltd., Tel Aviv 6473921, Israel; reza@redhillbio.com (R.F.); aida@redhillbio.com (A.B.); patricia.anderson@redhillbio.com (P.A.); 12 CEEF Solutions, Pointe-Claire, QC H9S 4L7, Canada; clara@redhillbio.com; 13 Middleburg Consultants, Takoma Park, MD 20912, USA; harris@middleburgconsultants.com; 14 Syneos Health, Morrisville, NC 27560, USA; shuhong.zhao@syneoshealth.com; 15 M2g Consulting, Inc., New York, NY 10024, USA; ikalfus@aol.com

**Keywords:** RHB-104, Crohn’s disease, *Mycobacterium avium* subspecies *paratuberculosis*, clinical trial, clarithromycin, rifabutin, clofazimine

## Abstract

This study, conducted between 4 October 2013, and 30 November 2018, tested the hypothesis that triple antimicrobial therapy, targeting *Mycobacterium avium* subspecies *paratuberculosis* (MAP), long considered a putative cause, would favorably affect Crohn’s disease. A double-blind multicenter study of adults with active Crohn’s disease, (i.e., Crohn’s Disease Activity Index [CDAI] 220–450 plus C-reactive protein ≥ 1.0 mg/dL, fecal calprotectin (FCP) >162.9 µg/g stool, or recent endoscopic or radiographic confirmation of active disease) receiving concomitant standard-of-care Crohn’s disease treatment (Clinicaltrials.gov: NCT01951326) were stratified by anti-tumor necrosis factor use and randomized (1:1) to anti-MAP RHB-104 (clarithromycin 95 mg, rifabutin 45 mg, and clofazimine 10 mg per capsule) (n = 166), resulting in clarithromycin 950 mg/day, rifabutin 450 mg/day, and clofazimine 100 mg/day, or placebo (n = 165) for up to 52 weeks. A greater proportion of RHB-104 versus placebo-treated patients met the primary endpoint—remission (i.e., CDAI < 150)—at week 26 (36.7% [61/166] vs. 22.4% [37/165], respectively; 95% CI for difference: 4.6, 24.0, *p* = 0.0048; chi-square test). Clinical response (reduction of CDAI by ≥100 points from baseline) at week 26 (first secondary endpoint) was also higher among the patients treated with RHB-104 (73/166 [44.0%]) compared with placebo (50/165 [30.3%]; 95% CI for difference: 3.4, 24.0, *p* = 0.0116), and it remained higher at week 52 among the patients treated with RHB-104 (59/166 [35.5%] vs. (35/165 [21.2%] for placebo; 95% CI for difference: 4.7, 23.9, *p* = 0.0042). A statistically significantly greater decline in FCP (another prospective efficacy endpoint) was also observed in RHB-104-treated patients, compared with placebo, at weeks 12, 26, and 52. The rates of serious adverse events were similar between groups (RHB-104: 18.7%; placebo: 18.8%). No patient died during the study. Antimicrobial therapy directed against MAP resulted in significantly greater improvement in clinical and laboratory (FCP) measures of active Crohn’s disease.

## 1. Introduction

Crohn’s disease is a chronic, recurrent, debilitating, and potentially life-threatening intestinal disease of unknown etiology and increasing prevalence [1]. Rather than targeting a specific etiology, current therapies for inflammatory bowel disease focus on immunosuppression to reduce inflammation. A wide variety of immunosuppressives have proven to be clinically effective compared to placebo, although remission rates remain modest [2]. For example, approximately two-thirds of anti-tumor necrosis factor (TNF) naïve patients exhibit a response after being treated with anti-TNF monoclonal antibodies, which is the most commonly used class of agents. In addition, approximately one-third of those who initially respond subsequently become non-responders or treatment intolerant [3,4,5].

The cause of Crohn’s disease remains unknown. The postulated pathogenesis includes immune dysregulation related to a genetic predisposition, microbial dysbiosis, and infections with specific microbial agents [6]. In 1913, the Scottish surgeon Dalziel linked Crohn’s disease to a similar illness in ruminants caused by infection with the *Mycobacterium avium* subspecies *paratuberculosis* (MAP) [7]. Since that time, MAP has been suspected of being involved in the etiology of Crohn’s disease. MAP infections are associated with the dysregulation of immune signaling pathways in immune cells and intestinal tissues [8], and the organism has been identified in over 50% of patients with Crohn’s disease, compared with 10 to 20% of controls [9,10,11]. Although research with immunosuppressives remains dominant, research regarding the association between MAP and Crohn’s disease has also remained active [12,13,14,15,16,17,18,19]. This association of MAP with Crohn’s disease has prompted experiments designed to test the hypothesis that, if MAP is etiologically involved in the pathogenesis of Crohn’s disease, then a treatment with antimicrobials designed to treat that mycobacterial infection would likely have a beneficial effect on the course and, eventually, possibly cure Crohn’s disease. Several studies with favorable results with anti-MAP therapies in patients with Crohn’s disease support this hypothesis [20,21] (e.g., Selby et al. randomized 213 patients with active Crohn’s disease to clarithromycin 750 mg/day, rifabutin 450 mg/day, and clofazimine 50 mg/day or matching placebos for 104 weeks, each combined with a 16-week tapering course of prednisolone at study entry [22]. Those in remission after 16 weeks continued participation in a maintenance phase. Significantly more patients receiving anti-MAP therapy than the placebo were in remission at week 16. However, the proportion who had relapsed by 52 weeks was not significantly different between the treatment groups (*p* = 0.054). This original interpretation of the study results as statistically nonsignificant was subsequently called into question based on the fact that non-remitters at week 16 had been excluded from the final analyses [23]. A reanalysis including the excluded patients showed significant results at week 52 (*p* = 0.003) and week 104 (*p* = 0.005) by an intention-to-treat analysis (see Discussion) [23].

Here, we report the findings from a 52-week, multicenter, randomized, placebo-controlled study (MAPUS) designed to test the hypothesis that a triple anti-MAP antimicrobial therapy would have a favorable effect on the course of Crohn’s disease. The drugs utilized were all previously confirmed to exhibit potent anti-MAP growth activity in vitro [24,25]. This study used a fixed dose combination of clarithromycin 950 mg/day, rifabutin 450 mg/day, and clofazimine 100 mg/day (RHB-104) or matching placebo in patients with moderately to severely active Crohn’s disease.

## 2. Results

**Figure 1:**
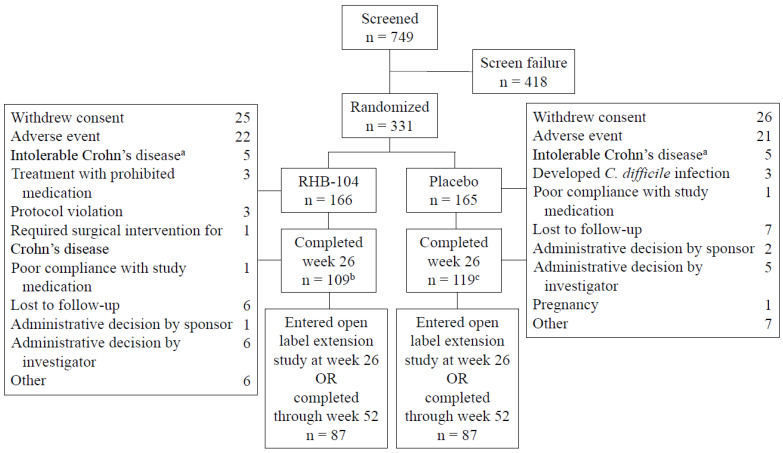
Patients flow through the study. ^a.^ Patients with symptoms of Crohn’s disease that led to their withdrawal from the study. ^b.^ Two patients completed week 26 but did not have Crohn’s Disease Activity Index (CDAI) measurements. ^c.^ One patient completed week 26 but did not have a CDAI measurement.

The study was conducted between 4 October 2013 and 30 November 2018. A total of 166 patients were randomized to RHB-104 and 165 to placebo. The treatment groups were balanced with respect to demographic and baseline clinical characteristics (Table 1). The mean (SD) patient age was 39.1 (12.5) years, and the mean (SD) time from diagnosis to randomization was 10.6 (9.0) years. The mean (SD) Crohn’s Disease Activity Index (CDAI) at baseline was 296 (55), and the primary site of the disease was the ileum, followed by the colon. The rate of ileal involvement was slightly higher among the patients in the RHB-104 group. The use of concomitant medications for Crohn’s disease was similar between the treatment groups, with 30% of patients using corticosteroids at baseline and 50% of patients using concomitant immunomodulators, including 24% of patients using concomitant anti-TNF agents. The disposition of patients is presented in Figure 1. One hundred and nine patients receiving RHB-104 completed week 26 compared to 119 patients receiving a placebo, with 87 in each treatment group entering the open-label study of RHB-104.

### 2.1. Primary Endpoint

**Figure 2:**
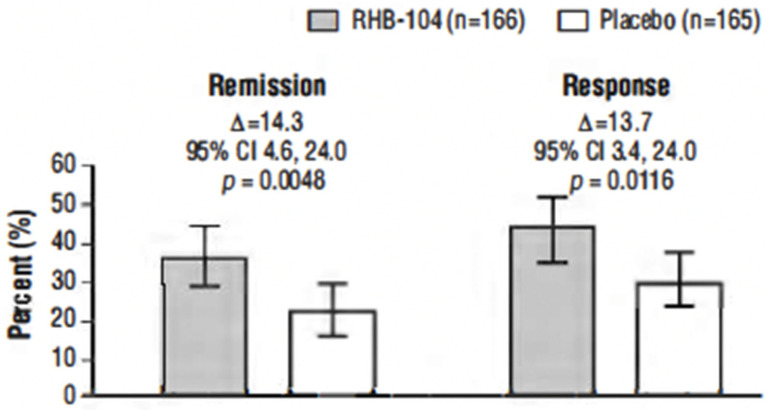
Clinical remission and clinical response at week 26. Notes: The primary efficacy endpoint was the proportion of patients who achieved clinical remission (Crohn’s Disease Activity Index [CDAI] score of <150) at week 26. The first key secondary endpoint was the proportion of patients who achieved clinical response at week 26 (defined as reduction of CDAI by ≥100 points from baseline to week 26). Analysis by Cochran–Mantel–Haenszel (CMH) chi-square test with stratification according to anti-TNF agents use.

**Figure 3:**
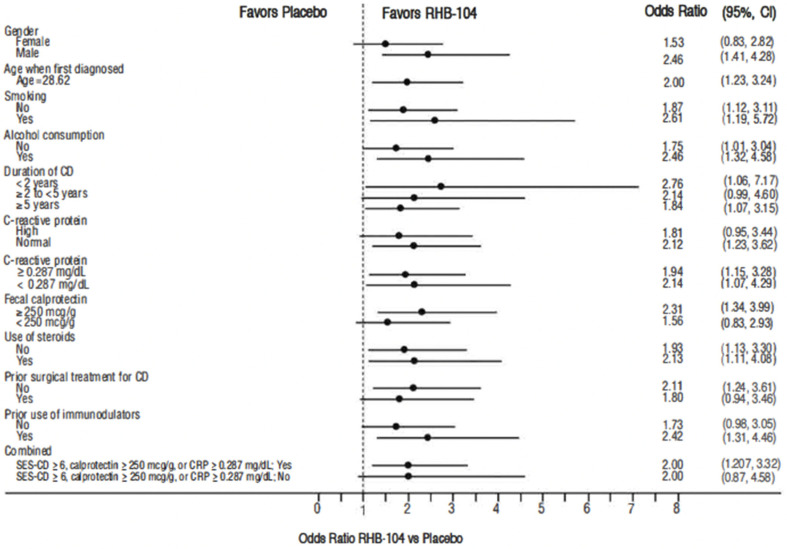
Forest plot for remission rate at week 26: odds ratio of RHB-104 versus placebo (95% CI) by subgroup. CD = Crohn’s disease; CI = confidence interval; CRP = C-reactive protein. Note: odds ratio of week 26 remission rate (RHB-104 vs. Placebo) and 95% Wald CI are based on adjusted logistic regression modeling with treatment, anti-TNF as fixed factors, and treatment by corresponding baseline-factor interaction term.

The primary endpoint, clinical remission at week 26, was met in 61/166 (36.7%) of patients who received RHB-104 vs. 37/165 (22.4%) of patients who received a placebo (∆14.3%; 95% CI for difference: 4.6, 24.0, *p* = 0.0048; Figure 2). In exploratory analyses of the primary endpoint using adjusted logistic regression modeling, a consistent treatment benefit favoring RHB-104 was observed across clinically important baseline factors/variables with prognostic potential (Figure 3).

### 2.2. Secondary Endpoints

The clinical response rate at week 26 was also significantly higher among the patients treated with RHB-104 (73/166 [44.0%]) compared with placebo (50/165 [30.3%]) (∆13.7%; *p* = 0.0116) (Figure 2). Likewise, the clinical response at week 52 (another prospective endpoint) remained higher among the patients treated with RHB-104 (59/166 [35.5%]) compared with placebo (35/165 [21.2%]) (95% CI for difference: 4.7, 23.9, *p* = 0.0042).

In prespecified hierarchical testing of the other key secondary endpoints, clinical remission at week 52 (the second key secondary endpoint) was numerically higher for the RHB-104 group compared to the placebo group (47/166 [28.3%] vs. 32/165 [19.4%]), but the difference was not significant (*p* = 0.0616; Table 2A). Importantly, the study was not powered for the remission week 52 endpoint, as an open-label study (RHB-104-04 study; ClinicalTrials.gov: NCT03009396) was instituted for those patients who failed to achieve remission at week 26, effectively limiting an evaluation of this endpoint. Furthermore, greater numbers of patients who received a placebo than those who received RHB-104 dropped out and entered the open-label study (Figure 1). Despite the lack of statistical significance on this second key secondary endpoint, the effect sizes for the remaining three key secondary endpoints of durable remission (between weeks 26–52), early remission at week 16, and corticosteroid-free remission (defined as CDAI <150 while maintained off steroids for at least 3 weeks) at week 52 all favored RHB-104 (Table 2A).

A higher proportion of patients receiving RHB-104 achieved the other prospective efficacy endpoint of clinical remission from week 16 through week 52 (18.7% vs. 8.5%, *p* = 0.008). In addition, a post hoc analysis of clinical remission at both weeks 16 and 52, which is consistent with published Crohn’s disease endpoints, favored RHB-104 (25.9% vs. 12.1%, *p* = 0.0016). Differences in the rates of clinical remission at week 26 between treatment groups favored RHB-104 versus placebo in patients taking concomitant anti-TNF agents (adalimumab and infliximab) and other immunomodulators including azathioprine and corticosteroids (Table 2B).

### 2.3. Other Efficacy Endpoints—PRO-2, FCP, CRP, and SES-CD Score

**Figure 4:**
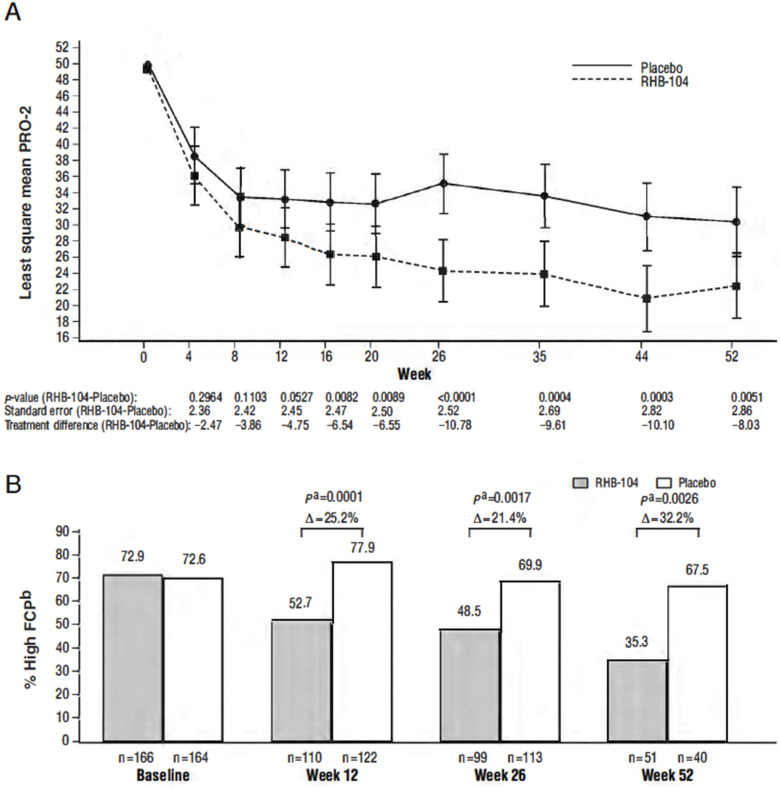
(**A**) Changes in Patient-Reported Outcome-2 Score over 52 weeks; (**B**) proportion of patients with elevated fecal calprotectin among those with an elevated baseline level. ^a^ Calculated with Cochran–Mantel–Haenszel (CMH) chi-square test, with stratification according to anti-TNF agents use. ^b^ High fecal calprotectin (FCP) defined as > 162.9 µg/g stool. Note: change in PRO-2 score over 52 weeks was analyzed by analysis of covariance adjusted for baseline values.

Differences in the PRO-2 (abdominal pain and loose-stool domains of the CDAI) between treatment groups favored RHB-104 as early as week 4 and thereafter, with the between-group difference statistically significant (*p* < 0.0089) at all visits from week 16 through week 52 (Figure 4A). Steady decreases favoring RHB-104 were also observed in the proportion of patients with elevated FCP (>162.9 µg/g stool) at baseline who continued to have elevated values at subsequent time points (Figure 4B), with the between-group difference statistically significant at weeks 12, 26, and 52. No difference was observed in the C-reactive protein (CRP) values post-treatment. While only a small subset of patients (n = 35) underwent a colonoscopy, a greater proportion of patients who received RHB-104 achieved 25% (RHB-104: 5/14 (35.7%) vs. placebo: 2/21 (9.5%), *p* = 0.0478) and 50% (4/14 [28.6%] vs. 1/21 [4.8%], *p* = 0.1076) decreases in the Simplified Endoscopic Activity Score for Crohn’s Disease (SES-CD) score at week 26.

### 2.4. Results in Patients With More Active Disease

In a post hoc analysis of the subpopulation of patients with more active disease, defined as FCP ≥ 250 µg/g stool, CRP ≥ 0.287 mg/dL, or SES-CD ≥ 6 at baseline, a greater proportion of patients receiving RHB-104 achieved clinical remission at week 52 compared to placebo (40/136 [29.4%] vs. 27/145 [18.6%], *p* = 0.0365). In addition, a greater proportion of patients with more active disease who received RHB-104 achieved clinical remission, with at least a 50% reduction from baseline in either the FCP or CRP concentration at week 16 (25/136 [18.4%] vs. 11/145 [7.6%], *p* = 0.0075), week 26 (36/136 [26.5%] vs. 7/145 [4.8%], *p <* 0.0001), and week 52 (31/136 [22.8%] vs. 11/145 [7.6%], *p* = 0.0004).

### 2.5. MAP Testing

The positive MAP buffy coat culture was similar for the active therapy and placebo groups at baseline (30.9% vs. 32.7%, week 26 (23.8% vs. 24.0%), and week 52 (23.9% vs. 34.7%). MAP cultures were more often positive in North America but remained similar between the active therapy and placebo treatment groups at baseline (50.0% vs. 55.1%), week 26 (43.1% vs. 41.6%), and week 52 (39.4% vs. 50.0%). The remission rates in baseline MAP-positive patients (34.1% vs. 21.4%, respectively; *p* = 0.2637) and in baseline MAP-negative patients (32.5% vs. 25.7%; *p* = 0.4727) were similar. Using a logistic regression model with fixed factors of treatment, anti-TNF, baseline MAP status, and treatment interaction term, no significant interaction was observed between MAP culture status at baseline (from buffy coats or colonic biopsy) and treatment effect on achieving remission at week 26 or 52. The *p*-values are 0.7861 and 0.3459 for the interaction term for weeks 26 and 52, respectively.

### 2.6. Compliance

RHB-104 was administered as five capsules twice a day, and compliance was monitored via returned pill counts at each visit. Over 91% of subjects in each study group (RHB-104: 91.7%, placebo: 91.5%) took over 80% of the planned medication.

### 2.7. Adverse Events

The type and incidence of adverse events reported over the 52-week treatment period were similar, in general, between the RHB-104 and placebo groups (Table 3). The incidences of chromaturia, a described effect of rifabutin, and nausea were higher among patients in the active treatment group than in the comparator group. There were no riboflavin-associated adverse effects noted in the study. The rate of *Clostridioides difficile* infection was lower in patients who received RHB-104, with 4 (2.4%) patients receiving RHB-104 developing infection vs. 14 (8.5%) of those receiving placebo. Only six (3.6%) patients receiving RHB-104 reported skin discoloration. A single case of uveitis, a reported complication of rifabutin, was observed in a placebo patient, but no cases were reported in patients receiving RHB-104.

Most adverse events were mild or moderate, with similar reporting in the active and control groups. Mild events occurred in 39.2% of the RHB-104 patients and 34.5% of the placebo patients. Moderate events occurred in 36.7% and 30.3% of the patients, and severe events occurred in 11.4% and 17.0% of the patients in the respective treatment groups.

Thirty-five (21.1%) and 31 (18.8%) patients in the active and control groups, respectively, experienced one or more adverse events leading to discontinuation of the study drug. The most common adverse events leading to discontinuation of the study drug were worsening Crohn’s disease (4.2% vs. 4.8%) and nausea (2.4% vs. 0.6%) (RHB-104 vs. placebo, respectively). The rates of serious adverse events were similar between the treatment groups (RHB-104: 18.7%; placebo: 18.8%). No patient died during the study.

### 2.8. Electrocardiogram Findings

A central electrocardiogram (ECG) analysis of QT interval data using Fridericia (QTcF) demonstrated an increased mean change from baseline QTcF (∆QTcF) across visits in patients receiving RHB-104, with the largest ΔQTcF of 29.8 ms at week 52. Mean placebo-corrected ΔQTcF increased from 2.8 ms (90% CI: −3.12 to 8.71) at week 1 to 30.6 ms (90% CI: 27.34 to 33.84) at week 52. There were 72 patients (66 on RHB-104, 6 on placebo), with ΔQTcF between 30 and 60 ms and 6 patients on RHB-104 with ΔQTcF > 60 ms. There were no patients with QTcF above 500 ms. In the drug concentration-QTc analysis, the clofazimine concentration appeared to be most relevant to QT prolongation, although clarithromycin contribution could not be excluded.

RHB-104 at the studied doses did not have a clinically relevant effect on cardiac conduction (i.e., the PR and QRS intervals). There were no PR or QRS outliers, except for one patient on active treatment with a QRS value above 110 ms and an increase in ΔQRS >  110 ms. In the diagnostic interpretation of the ECG findings, notable findings included a treatment-emergent first-degree atrioventricular block in three and five patients and a right bundle branch block in two and five patients on RHB-104 and a placebo, respectively.

## 3. Discussion

The study design was based on the hypothesis that infection with MAP is the primary cause of Crohn’s disease and that antimicrobial therapy designed to treat MAP would favorably influence the outcome of Crohn’s disease. MAP is a member of the *Mycobacterium avium* complex (MAC); infections with these organisms are notoriously difficult to cure, despite long-term therapy [26,27]. As such, a cure generally requires very prolonged therapy, is often elusive, and requires long-term suppressive therapy [27]. The combination of clarithromycin and rifabutin is currently approved in the US for the treatment of MAC in patients with advanced HIV infection. The study regimen added clofazimine, a potent anti-mycobacterial agent approved for the treatment of leprosy, to reduce the emergence of resistance and enhance the bactericidal effect [28]. The regimen proved effective, as it favorably influenced the outcome of Crohn’s disease compared to the effects evident in the population receiving TNFα inhibitors.

MAP is an obligate intracellular organism, as it lacks siderophores that bind or transport iron into cells [9]. Mycobacteria are thought to generally reside within host macrophages, where they may remain dormant or multiply very slowly [9,29]. Nonetheless, the presence of MAP provides an ongoing source of antigens to elicit an ongoing immune response that is theoretically manifested clinically as chronic inflammatory intestinal disease. MAP infection has also been shown to dysregulate immune signaling pathways by TNF and IFN-γ, which may enhance mycobacterial survival [30,31]. Hindrances to identifying MAP include its tendency to become cell-wall deficient, making microscopic detection difficult, and its unusually slow growth, making culture difficult [9]. In this study, buffy coat and biopsy samples have not been directly analyzed for MAP DNA due to the unavailability of a validated MAP diagnostic. These samples are being stored for future analysis. Further studies will also address the discrepancy between North American and ex-North American MAP culture results. Despite significant efforts extending beyond the completion of the clinical study, the development of a validated MAP diagnostic remains elusive and under investigation.

This study tested whether the addition of anti-MAP antimicrobial therapy (RHB-104) to standard-of-care therapy, including immunosuppressive therapy with TNF inhibitors, was superior to placebo plus standard-of-care for the therapy of patients with moderately to severely active Crohn’s disease. Compared to placebo, RHB-104 significantly improved the proportion of patients who achieved remission at both week 16 and the primary endpoint at week 26.

Prior studies have also tested the hypothesis that antimicrobial therapy directed toward MAP would elicit a beneficial effect in Crohn’s disease. These include a study conducted by Borody et al. almost two decades ago (up to a 54-month follow-up), in which 6 of 12 patients achieved full (clinical, colonoscopic, and histologic) remission and 2 additional patients achieved complete clinical remission with mild histological inflammation [32]. In a meta-analysis of 16 randomized clinical trials by Feller et al., the authors stated that “the benefit of some antibiotic regimens given for 3 months may be comparable to what is achieved with the anti–tumor necrosis factor alpha agents, with a potentially more favorable adverse effect profile and lower costs.” [26]. As noted in the Introduction, a previously conducted, randomized, placebo-controlled trial of anti-MAP therapy employing a similar antibiotic combination demonstrated significant differences in clinical remission at week 16, but there was a lack of significance on a per protocol basis at week 52 (*p* = 0.054), which was interpreted as a negative result [22]. However, reanalysis by Behr and Hanley, who included patients not in remission at week 16, showed significant results at weeks 52 (*p* = 0.003) and 104 (*p* = 0.005) by an intention-to-treat analysis [23].

MAP is one of the slowest-growing mycobacterial pathogens, and it is unknown how long treatment must be extended for control and, possibly, even an ultimate cure. The majority of antibacterial agents are most effective when the target microorganism is metabolically active, and thus, the treatment duration must be sufficiently long to not only kill the metabolically active bacteria but also the organisms when they re-activate or when patients are re-exposed to the infection [33,34]. TNF is essential for granuloma formation, which attempts to isolate and quarantine Mycobacterial infections. Conversely, it has also been suggested that anti-TNF agents may decrease MAP survival in macrophages [35]. Concomitant treatment with anti-TNF agents could facilitate the unmasking and killing of MAP infection by RHB-104, suggesting synergistic effects between anti-TNF and MAP therapy.

However, although the results point toward an etiologic role, by themselves they are insufficient to establish MAP as the cause of Crohn’s disease. Alternate hypotheses include the treatment of as yet unknown organisms or possibly unknown activities of the drugs used. It is important to note that each of the individual drugs employed in the RHB-104 combination, when examined as monotherapy in randomized controlled trials of three or more months, failed to show a benefit compared to placebo for Crohn’s disease (e.g., clarithromycin therapy [36,37], rifabutin [38], and clofazimine [39]). In each of these trials, a dose comparable to the quantity of each individual drug in RHB-104 was administered. Together these studies are consistent with the hypothesis that monotherapies using these antibiotics are as ineffective for Crohn’s disease as they are for *M. avium* infections.

Two unique features of this study were (1) patients were permitted concomitant treatment with infliximab and adalimumab at study entry (i.e., these medications were not disallowed or discontinued); and (2) corticosteroid tapering was started during the remission induction period and as early as week 8. To our knowledge, no other randomized, controlled induction-of-remission trial for Crohn’s disease has been conducted in which TNF agents could be continued throughout the treatment period.

In this study, the onset of symptom improvement by PRO-2 was observed as early as week 4, with statistically significant differences by week 16. Steady reductions in FCP were observed favoring RHB-104. Similar trends were observed in the small subset of patients who underwent a colonoscopy. The effect size with RHB-104 was greater in patients using concomitant therapy along with anti-TNF agents, azathioprine, and/or corticosteroids than without, while the overall response rates were similar. More importantly, we showed that RHB-104 could be used safely and effectively with concomitant anti-TNF agents, and it possibly enhanced the treatment response. Of note, the interesting finding regarding the lower rate of *C. difficile* infection in patients who received RHB-104 versus those receiving placebo could be related to improved mucosal health in the RHB-104-treated patients.

Certain limitations of the study merit comment. First, the enrollment criteria of CRP ≥ 1.0 mg/dL was high by the standards set in more recent Crohn’s disease trials of 0.287 mg/dL, while FCP ≥ 162.9 µg/g stool was low by those standards [40]. Second, no changes in CRP were noted, as was also the case in the vedolizumab pivotal study [40] and the Selby 2007 trial [22], suggesting that CRP may not be a sensitive marker of improving inflammation with certain treatments. Third, the imbalance of chromaturia could have resulted in the unblinding of patients and investigators. That said, other researchers [22] found that discoloration of urine did not unblind investigators, and when remission rates were reanalyzed in the current trial, the rate of remission among patients who reported chromaturia was similar to that of the general study population. Fourth, the diagnostic methods for MAP have not been standardized, which could affect the interpretation of the study results. Finally, patient follow-up was limited to a maximum of 52 weeks and only a subset of patients (n = 75) received RHB-104 for that long. More patients who received a placebo failed to achieve remission at week 26 and entered the open-label extension, creating a bias at week 52 that favored placebo. More information is needed about the durability of the effect in patients who achieve remission at the completion of treatment and about the need for a much longer duration of therapy. Furthermore, more detailed data over a longer follow-up period will further inform on long-term drug safety.

Since the inception and approval of this study, the endpoints for studies of Crohn’s disease have been repeatedly reassessed, with mucosal healing emerging as one of the preferred outcomes. As such, this study did not include mucosal healing but did include a surrogate, FCP, the results for which were supportive of a clinical effect.

In summary, treatment with RHB-104, consisting of a fixed-dose combination of antibiotics chosen for their effectiveness in *M. avium paratuberculosis* infection, appears to be effective for the treatment of Crohn’s disease. We realize that non-antibacterial mechanisms of action, such as immune modulation, may be present. Although the advent of an accurate and reliable MAP diagnostic may help clarify the situation, an oral, safe, and effective therapy for CD would be highly beneficial to the patients and treating community. The triple anti-MAP therapy proved beneficial to patients receiving corticosteroids, immunosuppressive agents, or anti-TNF agents and may have a role as an add-on therapy for patients not responding to their current treatment. Additional studies are needed to establish a mechanism of action, clinical relevance and impact of QT prolongation, ideal duration of treatment, durability of response, and effect on mucosal healing. The role of antibiotics in the treatment of Crohn’s disease and the impact of the microbiome continue to be fertile areas of research, as we pursue methods to improve remission rates in this disease.

## 4. Materials and Methods

### 4.1. Ethical Practices and Study Registration

The study was conducted in accordance with the ethical principles of the Declaration of Helsinki and applicable regulatory requirements. The study protocol and all amendments were approved by the respective institutional review boards or ethics committees of the participating institutions. All patients were provided with a written informed consent form before participating in the study. The study was registered at Clinicaltrials.gov as NCT01951326.

### 4.2. Study Population

Eligible participants were between the ages of 18 to 75 years with moderately to severely active Crohn’s disease, defined as per the CDAI between 220 and 450, inclusive, and at least one of the following at screening: C-reactive protein (CRP) ≥ 1.0 mg/dL (the upper limit of normal of the reference lab), an FCP exceeding 162.9 µg/g stool (the upper limit of normal of the reference lab), or an endoscopic or radiographic (by magnetic resonance or computer tomographic enterography) confirmation of active Crohn’s disease in the prior 5 weeks. The endoscopic or radiographic presence of Crohn’s disease was determined by the local investigator, and specific findings were not required for study enrollment. These criteria are consistent with the inclusion criteria for Crohn’s disease studies conducted at the time of study design (2012).

A history of ileocolonic Crohn’s disease, diagnosed by endoscopy, radiography, and/or histology at least 6 months prior to randomization into the study was required. Patients must also have been receiving treatment with one of the following medications: oral 5-aminosalicylic acid (5-ASA) at a stable dose for 4 or more weeks, corticosteroid at a stable dose for 2 or more weeks, azathioprine, 6-mercaptopurine, or methotrexate at a stable dose for at least 8 weeks, and/or infliximab or adalimumab at a stable dose or had been discontinued for at least 14 weeks.

The key exclusion criteria included symptomatic stenosis or ileal strictures that might require surgery in the ensuing 12 months; a history of total colectomy with ileorectal anastomosis or a proctocolectomy; the presence of active fistulizing Crohn’s disease or healed fistula within 2 months prior to screening; postoperative stoma, ostomy, or ileoanal pouch; the presence of short bowel syndrome; surgical bowel resection scheduled; and known symptomatic obstructive strictures or bowel perforation in the 6 months prior to screening. Patients with a history of an atypical mycobacterial infection (other than MAP), or active tuberculosis requiring treatment in the past 3 years were also excluded, as were those who had received oral or parenteral antibiotics in the 4 weeks prior to baseline.

### 4.3. Study Design

This was a randomized, double-blind, placebo-controlled study conducted at 92 sites in the United States, Canada, Bulgaria, the Czech Republic, Australia, New Zealand, Israel, Poland, Serbia, and Slovakia.

### 4.4. Randomization and Blinding

The patients were stratified by concomitant anti-TNF agent use and, then, randomized (1:1) to receive treatment with RHB-104 or a placebo for up to 52 weeks. Importantly, all patients were required to receive concomitant standard-of-care Crohn’s disease treatment. Randomization was based on a centralized, computer-generated randomization schedule using randomly permuted blocks. Patients, study staff, and investigators/site personnel were blinded to treatment group assignment.

Each capsule of RHB-104 contained clarithromycin 95 mg, rifabutin 45 mg, and pegylated clofazimine 10 mg. The RHB-104 and placebo capsules were manufactured to be identical in appearance, with dark opaque capsules used to camouflage the characteristic coloration of clofazimine and rifabutin. Riboflavin, 25 mg, was added to the placebo formulation to mimic changes in urinary coloration from rifabutin.

### 4.5. Study Drug Dosing and Concomitant Medications

To maximize the tolerability of the multidrug multiple dose strategy, dosing of the study drug began as 1 capsule twice daily with food and was increased by 1 capsule twice daily each week over the first 5 weeks of the study to achieve a dose of 5 capsules twice daily at week 5, resulting in a total daily dose of clarithromycin 950 mg, rifabutin 450 mg, and clofazimine 100 mg from week 5 through to week 52, or the conclusion of a patient’s participation in the trial. The patients who completed 26 weeks of the study drug and did not achieve remission at week 26 [defined as a persistent CDAI ≥150] were eligible to enter an open-label extension study of RHB-104 (NCT03009396), during which the original treatment assignment remained blinded. These patients were treated for 52 weeks with open-label RHB-104.

Concomitant standard-of-care therapy for Crohn’s disease was maintained at stable doses throughout the course of the study, as described above, with the exception of corticosteroids, which, at the discretion of the investigator, could be tapered beginning at week 8. Other biologic agents were prohibited from screening through week 52.

Patients with new or unresolved Crohn’s disease symptoms could receive rescue therapy with oral, rectal, or systemic therapies, including 5-ASA compounds, immunosuppressives, corticosteroids, anti-TNF agents, or prohibited antibiotics (i.e., might have drug–drug interaction with study drug), and remain on the study drug at the discretion of the investigator. These patients were considered treatment failures.

### 4.6. Efficacy and Safety Assessments

Patients completed a diary on a daily basis for the 7 days prior to baseline and the week 4, 8, 12, 16, 20, 26, 35, 44, and 52 visits for the calculation of CDAI. The serum CRP level and the FCP were determined at the baseline and post-baseline visits. Colonoscopy was optional and was performed on consenting patients prior to initiation of the study drug and at week 26. Endoscopic disease activity was assessed at baseline and post-baseline timepoints by a single-blinded central reader using the SES-CD [41]. An assay for MAP was performed on DNA isolated from cultures of both buffy coat and colon tissue (as available) using a sensitive and specific nested PCR assay (University of Central Florida (UCF), Orlando, FL, USA) [10]. North American samples were processed entirely at UCF while ex-North American samples were initially processed to buffy coat and then shipped to UCF.

Adverse events and other safety assessments (i.e., hematology and serum chemistry, urinalysis, physical examination, and uveitis assessment) were monitored throughout the study, including at a visit conducted 4 weeks post-treatment. ECGs were performed at screening, baseline, and the weeks 2, 4, 8, 12, 26, and 52 visits. An independent Data Safety Monitoring Board (DSMB) monitored safety data over the course of the trial. The DSMB also reviewed data from a pre-specified interim analysis for safety and futility after half of the patients had completed 26 weeks of treatment. Regarding the interim efficacy (or inefficacy) of the data, recommendations for early termination were guided by the Lan-DeMets alpha-spending implementation of the O’Brien–Fleming spending function to determine the test boundaries. The efficacy boundary preserved the overall 2-sided study alpha level of 0.05. The alpha level at the first and second sequential tests was 0.003 and 0.049, respectively. Upon review of the safety and efficacy data, the DSMB recommended that the study be continued without changes to the protocol or study conduct.

### 4.7. Statistical Methods

#### 4.7.1. Efficacy Endpoints and Analyses

The primary efficacy endpoint was the proportion of patients who achieved clinical remission (CDAI score of <150) at week 26. Patients who did not achieve remission at week 26 (CDAI ≥ 150), did not have a CDAI measurement at week 26, or received rescue medications were considered treatment failures in this intent-to-treat analysis.

Key secondary endpoints included (1) response at week 26 (defined as a reduction of CDAI by ≥100 points from baseline to week 26); (2) remission at week 52; (3) durable remission (from week 26 through week 52 continuously); (4) remission at week 16; and (5) corticosteroid-free remission at week 52. Patients who did not achieve remission at week 52 (CDAI ≥ 150), did not have a CDAI measurement at week 52, or received rescue medications were considered treatment failures in this intent-to-treat analysis.

A serial gatekeeping (fixed sequence) approach was applied to adjust for multiplicity and to control type-1 error across the primary endpoint and secondary efficacy endpoints. Therefore, the endpoints were analyzed sequentially and were only considered significant if the endpoint individually and the previous endpoints in the hierarchy, including the primary endpoint, were also significant at the 2-sided 0.049 level.

Other prospective efficacy endpoints included remission from week 16 through week 52; remission at week 26 in the subgroups receiving concomitant corticosteroids or immunomodulators, including azathioprine and anti-TNF agents; response at week 52; changes in CRP and FCP at study visits; and the proportion of patients who were endoscopic responders, defined as a 25% or 50% reduction in the SES-CD score from baseline to week 26. Post hoc endpoints include induction of clinical remission at week 16 with maintenance at week 52, which is consistent with approved Crohn’s disease therapies [40,42,43]; change in Patient-Reported Outcome-2 (PRO-2; the abdominal pain and loose stool domains of the CDAI) at study visits; and analyses related to inflammatory markers such as CRP [44] or FCP. Changes in MAP status via buffy coat PCR and buffy coat culture, as well as in those patients undergoing colonoscopy, were planned.

#### 4.7.2. Sample-Size Determination

The study was originally powered to 410 patients. However, based on the blinded pooled Week 26 remission rate of the first 222 patients enrolled (31%) and historical placebo remission rates (15%), the sample size was subsequently adjusted to 330 patients (165 per treatment arm) based on a blinded sample-size re-estimation. The final sample size was estimated to provide over 80% power at a 2-sided alpha of 0.049 to detect a 15% difference, assuming treatment effects in the active and placebo groups of 36% and 21%, respectively, for clinical remission at week 26.

#### 4.7.3. Statistical Methods

The data were analyzed based on analysis sets that included all randomized patients (efficacy) who received at least 1 dose of the study drug (safety). Statistical tests were conducted at a 2-sided 0.049 significance level. Analyses were performed using SAS, version 9.4.

Categorical endpoints were analyzed using the Cochran–Mantel–Haenszel (CMH) chi-square test, controlling for the stratification variable of anti-TNF agent use, and continuous endpoints were analyzed using an analysis of covariance (ANCOVA) with the baseline value as a covariate. Confidence intervals (CI) for categorical data were calculated from normal approximation.

Subgroup analyses on the primary endpoint were conducted using adjusted logistic regression modeling with treatment and anti-TNF therapy (adalimumab and infliximab) as fixed factors and the corresponding baseline-factor interaction term.

Putative modifiers included sex, age when first diagnosed with Crohn’s disease, smoking status, alcohol consumption status, duration of Crohn’s disease, prior use of immunomodulators, use of corticosteroids, prior surgical treatment of Crohn’s disease, baseline CRP level, baseline fecal calprotectin level, and the combination of FCP ≥ 250 µg/g stool, CRP ≥ 0.287 mg/dL, or SES-CD ≥ 6 at baseline. Fisher’s exact test was used to analyze the post hoc categorical endpoints of clinical remission.

Adverse events were analyzed using descriptive statistics. A drug concentration-QTc analysis was performed. A full model including clarithromycin, 14-OH-clarithromycin, rifabutin, 25-O-desacetyl-rifabutin, and clofazimine was initially fit, and a model selection procedure was performed. The model with clofazimine was selected and represented the data reasonably well. In the Bayesian post hoc analysis, the estimated population slope of the clofazimine plasma concentration-QTc relationship was 0.037 ms per ng/mL (90% CI: 0.0337 to 0.0410), with a treatment effect–specific intercept of 2.52 ms (90% CI: 0.76 to 4.29). The effect on ∆ΔQTcF can be predicted as a 28.8 ms (90% CI: 26.33 to 31.23) increase at the highest mean clofazimine concentration at week 52, which corresponds well to the observed QTc effect.

## Figures and Tables

**Table 1:** Demographic and baseline characteristics.

	RHB-104 N = 166	Placebo N = 165	Total N = 331
Gender, n (%)			
Male	91 (55)	98 (59)	189 (57)
Female	75 (45)	67 (41)	142 (43)
Age (years)			
Mean (SD)	39.0 (12.5)	39.3 (12.6)	39.1 (12.5)
Smoking status, n (%)			
Yes	33 (20)	30 (18)	63 (19)
No	133 (80)	135 (82)	268 (81)
Time from diagnosis to randomization, years			
Mean (SD)	10.4 (9.0)	10.8 (9.0)	10.6 (9.0)
Distribution, n (%)			
0 – < 2	20 (12.0)	18 (10.9)	38 (11.5)
2 – < 5	36 (21.7)	37 (22.4)	73 (22.1)
≥5	110 (66.3)	110 (66.7)	220 (66.5)
Site of primary diagnosis, n (%)			
Ileum	125 (75.3)	98 (59.4)	223 (67.4)
Colon	93 (56.0)	106 (64.2)	199 (60.1)
Other	12 (7.2)	8 (4.8)	20 (6.0)
CDAI			
Mean (SD)	298 (57)	293 (53)	296 (55)
CRP, mg/dL			
Mean (SD)	1.34 (1.75)	1.38 (1.87)	1.36 (1.81)
Calprotectin, µg/g stool			
Mean (SD)	543 (604)	668 (952)	605 (797)
Concomitant CD medications, n (%)			
Corticosteroids ^a^	50 (30.1)	50 (30.3)	100(30.3)
Immunomodulators ^b^	75 (45)	89 (54)	164 (50)
Adalimumab or infliximab	37 (22.3)	41 (24.9)	78 (24)

CD = Crohn’s disease; CDAI = Crohn’s Disease Activity Index; CRP = C-reactive protein; N = number of patients randomized to the treatment group or the total number of randomized patients; n = number of patients by characteristic within each treatment group or in the combined treatment groups; ^a^ patients receiving corticosteroids at baseline for Crohn’s disease; ^b^ included 6-mercaptopurine and immunosuppressants (e.g., azathioprine).

**Table 2:** (A). Secondary endpoints: clinical remission (intention-to-treat population). (B). Clinical remission at Week 26 per use (or not) of concomitant CD medications—anti-TNF agents, azathioprine, and corticosteroids.

(**A**) Secondary Endpoints: Clinical Remission (intent-to-treat population)					
			**Between-Group Comparison** **(RHB-104-Placebo)**		
**Endpoint**	**RHB-104**	**Placebo**	**Difference**	**95% CI**	***p*-Value**
Remission at week 52	47/166 (28.3%)	32/165 (19.4%)	8.9	−0.2, 18.0	0.0616
Durable remission weeks 26–52	33/166 (19.9%)	21/165 (12.7%)	7.2	−0.8, 15.1	0.0851
Remission at week 16	70/166 (42.2%)	48/165 (29.1%)	13.1	2.9, 23.3	0.0147
Corticosteroid-free remission at week 52	42/166(25.3)	29/165 (17.6)	7.7	−1.1, 16.5	0.0922
CI = confidence interval; Note: Remission is defined as Crohn’s Disease Activity Index (CDAI) < 150.					
(**B**) Remission at Week 26 per Use (or Not) of Concomitant CD Medications —anti-TNF Agents, Azathioprine, and Corticosteroids					
**Concomitant CD Medication**	**RHB-104**		**Placebo**		***p*-Value**
Adalimumab or infliximab agents					
Yes	11/31 (35.5%)		6/36 (16.7%)		0.0798 ^a^
No	50/135 (37.0%)		31/129 (24.0%)		0.0222 ^a^
Azathioprine					
Yes	17/37 (45.9%)		11/45 (24.4%)		0.0439 ^b^
No	44/129 (34.1%)		26/120 (21.7%)		0.0342 ^b^
Corticosteroids					
Yes	18/50 (36.0%)		7/50 (14.0%)		0.0134 ^b^
No	43/116 (37.1%)		30/115 (26.1%)		0.0753 ^b^

CD = Crohn’s disease; TNF = tumor necrosis factor. ^a^ *p*-values calculated using the Mantel–Haenszel (MH) chi-square test. ^b^ *p*-values calculated using the Cochran–Mantel–Haenszel chi-square test with stratification according to anti-TNF agents use. Note: Remission is defined as Crohn’s Disease Activity Index (CDAI) < 150. Values represent the number of patients meeting endpoint criteria divided by the number of patients in total. Numbers in parentheses are percentages.

**Table 3:** Most frequently reported adverse events.

	Number (%) of Patients			
Adverse Event	RHB-104 N = 166		Placebo N = 165	
Chromaturia	42	(25.3%)	2	(1.2%)
Abdominal pain	24	(14.5%)	19	(11.5%)
Nausea	22	(13.3%)	12	(7.3%)
Crohn’s disease worsening	21	(12.7%)	25	(15.2%)
Headache	16	(9.6%)	17	(10.3%)
Arthralgia	16	(9.6%)	7	(4.2%)
Vomiting	12	(7.2%)	7	(4.2%)
Diarrhea	11	(6.6%)	8	(4.8%)
Anemia	10	(6.0%)	6	(3.6%)
Pyrexia	9	(5.4%)	6	(3.6%)
Upper respiratory infection	8	(4.8%)	9	(5.5%)
Viral upper respiratory infection	7	(4.2%)	9	(5.5%)
Influenza	6	(3.6%)	10	(6.1%)
Abdominal tenderness	4	(2.4%)	9	(5.5%)
*Clostridioides difficile* infection ^a^	4	(2.4%)	14	(8.5%)

^a^ Taxonomic reclassification of a combination of 2 previously preferred terms, namely “*Clostridium difficile* infection” and “*Clostridium* test positive”. Notes: Adverse events are reported according to events with incidence ≥5% in either treatment group and presented in descending order of incidence within the RHB-104 group. Adverse events were reported from baseline through week 52, excluding patients during their participation in the open-label study.

## Data Availability

The study protocol, statistical analysis plan, informed consent form, and data collected for this study, including deidentified individual participant data and a data dictionary defining each field in the set, will be made available through the corresponding author with the publication of this paper.
